# Elucidation of the more myopic eye in anisometropia: the interplay of laterality, ocular dominance, and anisometropic magnitude

**DOI:** 10.1038/s41598-019-45996-1

**Published:** 2019-07-03

**Authors:** Siyu Jiang, Zheyi Chen, Hua Bi, Ruijing Xia, Ting Shen, Ling Zhou, Jun Jiang, Bin Zhang, Fan Lu

**Affiliations:** 1 Wenzhou Medical University, School of Ophthalmology and Optometry, Wenzhou, Zhejiang 325027 China; 2 Eye Hospital of Wenzhou Medical University, Wenzhou, Zhejiang 325027 China; 3 Nova Southeastern University, College of Optometry, Fort Lauderdale, FL 33314 USA

**Keywords:** Medical research, Neuroscience

## Abstract

This study reveals how, in a myopic anisometrope, the odds of an eye being more myopic are related to laterality, ocular dominance, and magnitude of anisometropia. In 193 subjects, objective refraction was performed with cycloplegia. Sighting, motor, and sensory dominance were determined with the hole-in-the-card test, convergence near-point test, continuous flashing technique, respectively. Multiple logistic regression was used for probability analysis. Seventy percent of the subjects had a right eye that was more myopic, while 30% of them had a more myopic left eye. When the right eye was the sensory dominant eye, the probability of the right eye being more myopic increased to 80% if the anisometropia was less than 3.0 D, and decreased below 70% if anisometropia was beyond 3.0 D. When the left eye was the sensory dominant eye, the probability of the left eye being more myopic increased to above 40% if the anisometropia was less than 4.0 D and decreased below 30% if the anisometropia was beyond 4.0 D. Therefore, between the two eyes of anisometropes, laterality tilts the chance of being more myopic to the right. Being the sensory dominant eye increases an eye’s probability of being more myopic by another 10% if the magnitude of anisometropia is moderate.

## Introduction

Anisometropia refers to the condition of unequal refractive errors between the two eyes, typically because of an interocular difference in axial lengths^1^. If an interocular difference in spherical equivalent (SE) refractive error greater than or equal to one diopter (1 D) is employed as the criterion, anisometropia affects around 10% of the population in early adulthood^2^. Myopic anisometropia is about two to five times more prevalent than hyperopic anisometropia^3^. The emmetropization failure underlying anisometropia and ametropia may be similar based on the findings from the following studies. The prevalence and magnitude of anisometropia are significantly associated with the refractive error of the less ametropic eye^4,5^. The prevalence, which is stable around 5% from one year of age to teenage, starts to rise with the onset of myopia^5–7^. Moreover, a longitudinal study shows that each eye of anisometropic children has a higher rate of myopic progression than isometropic controls^8^. Therefore, anisometropia could be used as a unique experimental paradigm in studying myopia development. While traditional cohort studies, which compare myopes with emmetropes, are often influenced by many potential confounding factors, e.g., age, gender, and environment, the comparison between the fellow eyes of the same anisometropic subjects provides better control for those factors.

Indeed, many studies have examined interocular asymmetry to identify elements leading to the emmetropization failure. The visual system is composed of both optic and neural components. Anisometropic eyes often display a high degree of interocular symmetry for a range of biometric and optical characteristics, including corneal power^9^, corneal resistance and hysteresis^10^, intraocular pressure^11^, corneal aberrations^10,12^, total ocular aberrations^9,13^, accommodative response^10,14^, and visual acuity^10,14^, although there are occasional reports of interocular difference in corneal power^10^ or toricity^15,16^. However, there have been many fewer studies investigating neural asymmetry in the anisometropic visual pathway even though interocular optical difference cannot fully account for the behavioral performance in anisometropes. For example, even with substantial anisometropia (−10 D), more than 50% of the myopic anisometropes retain stereoacuity of better than 40 arc sec.^17^, while a simulated anisometropia of even smaller magnitude would have severely impaired one’s stereoacuity^18,19^. Only in recent years have more studies started to explore the association between anisometropia and neural asymmetry, such as ocular dominance, which is the preference for using one eye’s information over the other^20,21^.

An often-asked question about myopic anisometropia is whether the dominant eye is more myopic or less myopic. Findings from previous studies are contradictory to each other, mainly due to the discrepancies on the following three key issues. The first one is the laterality, in which the right eye tends to be more myopic. Singh *et al*. reported a predisposition toward greater myopia in the right eye in 24 out of 31 anisometropes^22^. In Chia *et al*.’s study, axial length in the right eyes was found to be significantly longer than that in the left eyes although the SE was reported to be the same between the two eyes^23^. Goldschmidt *et al*. reported a tendency toward a higher amount of spherical myopia in the right eye in Hong Kong myopic anisometropic children and their parents^4^. Similar results have also been reported in other studies^24^. Therefore, to explore if the dominant eye tends to be more myopic, the effect of laterality has to be controlled.

The second is the methods used to measure ocular dominance. Different methods often lead to completely different conclusions. In most studies, ocular dominance is evaluated with respect to sighting dominance, which is often measured with the hole-in-the-card test. Using this method, some studies in adult populations revealed that the dominant eye is more myopic and has a longer axial length^25^, while others reported that the dominant eye is less myopic and has less astigmatism^26–28^. Other investigations suggested the absence of a significant association between ocular dominance and anisometropia^23,29,30^. Motor dominance, tested with the convergence near-point test, has been reported as not associated with the tendency of being more myopic^25^. More recent research on sensory dominance has reported that the dominant eyes were more myopic in myopic anisometropes and less hyperopic in hyperopic anisometropes^31^.

The third factor is the magnitude of the anisometropia. The findings of the studies are often valid only in anisometropes with certain magnitude. With a criterion of interocular SE difference larger than or equal to −0.5 D, Chia reported no significant association between ocular dominance and which eye is more myopic^23^. Cheng *et al*.’s study identified a threshold level of anisometropia of −1.75 D. In subjects with anisometropia beyond this level, the dominant eye is always more myopic^25^. In Vincent’s study, when anisometropia exceeded −1.75 D (dashed line) the dominant eye was the more myopic eye in 90% of subjects. When anisometropia was greater than −2.25 D, the dominant eye was always the more myopic eye^10^. Linked *et al*. reported that the nondominant eye is more myopic in the anisometropes with a criterion of −2.5 D^27^.

In previous studies, often just one or, at most, two factors were taken into consideration to derive a conclusion. As such, this might partially explain the inconsistency among the reports. In the present study, we reconsider the following question from a probabilistic approach, what are the odds that an eye is more myopic given its laterality, status of being the dominant eye, and the magnitude of anisometropia? The goal of the present study was to clarify the interplay of those three factors in determining which eye is more myopic via an analysis based on multiple logistic regression. We believe the findings from this study will help us understand the development of anisometropia better, and potentially provides new insights on myopic progress.

## Results

### Refractive errors

The refractive errors of the right and left eyes are summarized in Table 1. The right eyes showed significantly greater spherical power and SE than the left eyes (p < 0.001). However, there was no significant difference in cylinder power between the two eyes.

**Table 1:** Refractive errors of the right and left eye.

	Right eye		Left eye		Stats
	Mean ± SD	Median	Mean ± SD	Median	p value
Spherical	−3.79 ± 2.33	−3.75	−2.88 ± 2.47	−2.50	<0.001
Cylinder	−0.59 ± 0.60	−0.50	−0.66 ± 0.64	−0.50	0.276
SE	−4.09 ± 2.44	−3.75	−3.21 ± 2.55	−2.75	<0.001
J0	0.18 ± 0.33	0.12	0.21 ± 0.33	0.17	0.335
J45	0.03 ± 0.20	0.00	−0.06 ± 0.24	0.00	0.001

### Laterality: right vs left

**Figure 1:**
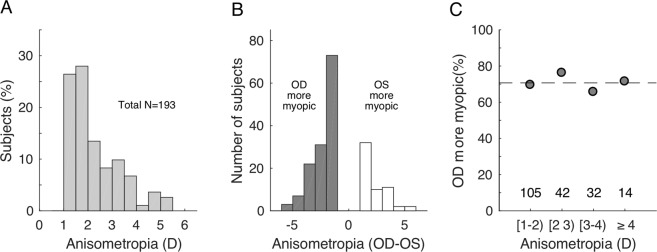
Anisometropia. (**A**) Distribution of the degrees of anisometropia. (**B**) Distribution of subjects with the right eye being more myopic (gray bars) or the left eye being more myopic with the degree of anisometropia. **C)** The percentages of right eyes being more myopic plotted against the degree of anisometropia.

More than half of the anisometropic subjects had anisometropia of less than 2D (105/193; 54.4%). Subjects became fewer in number as the degree of anisometropia increased (Fig. 1A). The right eye was more myopic in 136 subjects and the left eye was more myopic in 57 subjects (Fig. 1B). The percentage of right eyes that were more myopic remained constant at around 70%, across different ranges of anisometropia (Fig. 1C).

### Ocular dominance

Each subject was tested for sighting dominance, motor dominance, and sensory dominance. The percentages of subjects with two balanced eyes, with a dominant right eye, and with a dominant left eye are summarized in Table 2. For sighting dominance, the dominant eye was more myopic in slightly less than half of the subjects. In the motor and sensory dominance tests, the dominant eye was more myopic in more than half of the subjects (Table 2).

**Table 2:** Different types of ocular dominance tests.

	Balanced (n)	Right Dominant (n)	Left Dominant (n)	Right or left Dominant (n)	Dominant eye more myopic (n)
Sighting	0	102	91	193	95
Motor	56	74	63	137	74
Sensory	40	74	79	153	87

For an eye, given its laterality and whether it is the dominant eye, the probability of a certain eye being more myopic was regressed on two factors, laterality and being the dominant eye, simultaneously. After controlling for the effect of laterality, only ocular sensory dominance was significantly related. Ocular sighting and motor dominance have no effect after the influence of laterality was controlled (Table 3).

**Table 3:** Results of the multiple logistic regression test.

Formula: Odds of being myopic ~ Laterality + The status of being the dominant eye		
	**Laterality**	**The status of being the dominant eye**
Sighting	p < 0.001	p = 0.402
Motor	p < 0.001	p = 0.359
Sensory	p < 0.001	p = 0.002

### Ocular sensory dominance and the degree of anisometropia

**Figure 2:**
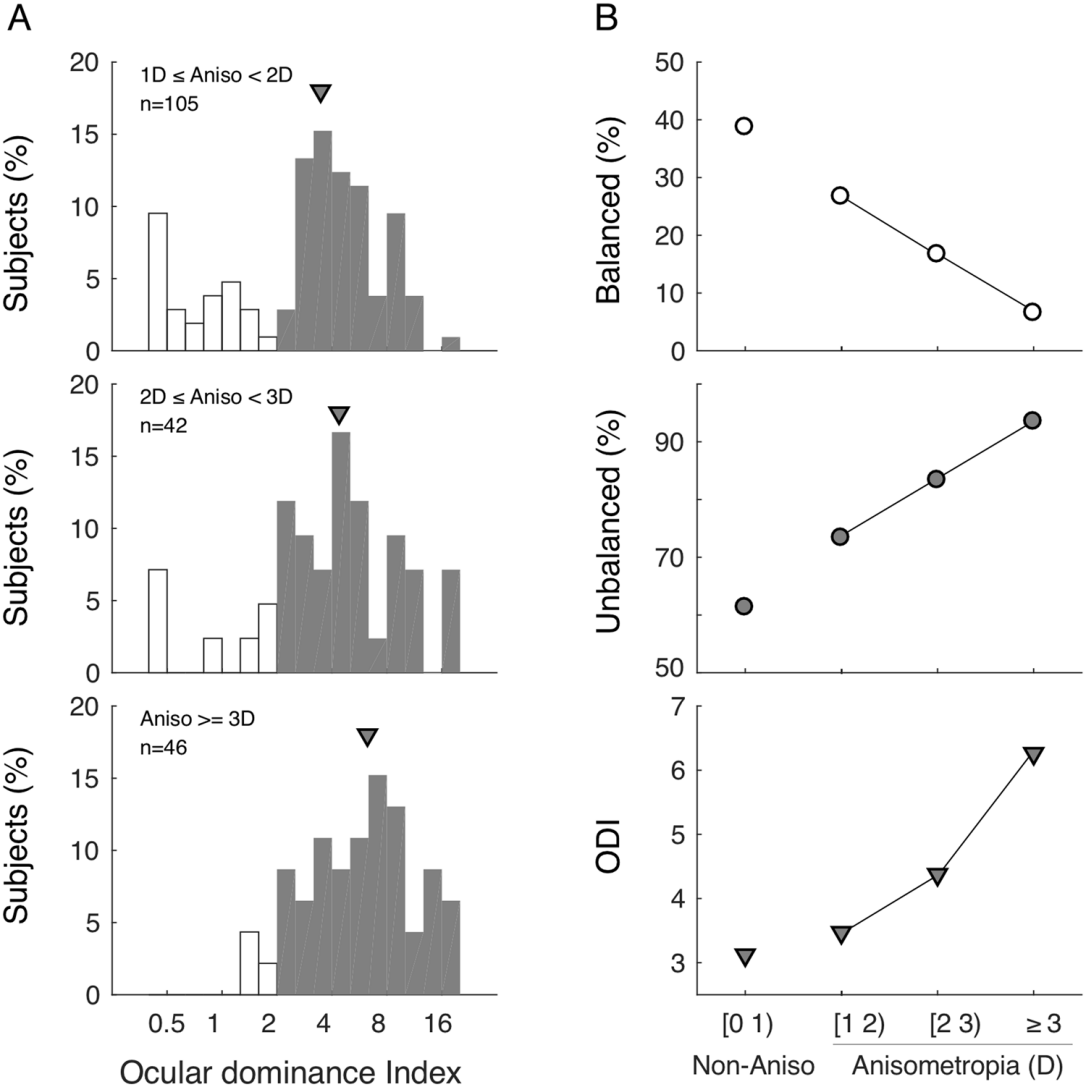
Association between ocular dominance and anisometropia. (**A**) The distribution of ODI in different ranges of anisometropia. The open bars represent subjects with balanced eyes and the filled bars represent subjects with unbalanced eyes. (**B**) Systemic changes in ocular dominance with the degree of anisometropia. The values of non-anisometropic subjects were obtained from Jiang *et al*. with permission.

In 193 anisometropic subjects, 20.2% of them had balanced eyes and 79.8% of them had clear sensory dominance, which is significantly higher than that in non-anisometropic subjects (61.3%^31^). In further analysis, subjects were separated into different groups according to the magnitude of anisometropia. As the degree of anisometropia became greater, the percentage of subjects with clear dominance increased, and the median ocular dominance index (ODI) value increased (Fig. 2).

### Combining the three factors together

**Figure 3:**
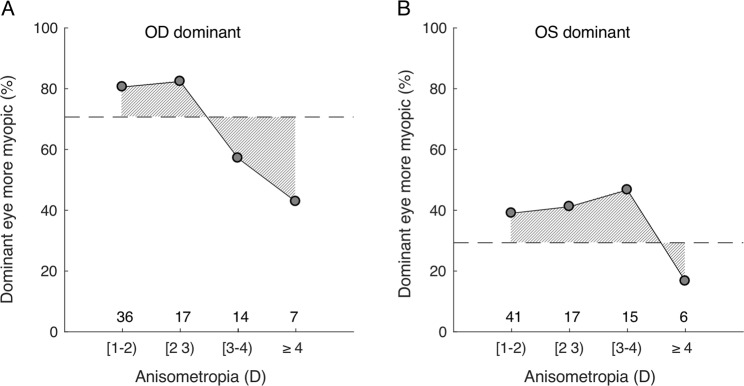
The percentage of the dominant eyes being more myopic vs the degree of anisometropia. (**A**) Subjects with the right eye as the dominant eye. (**B**) Subjects with the left eye as the dominant eye.

Without considering ocular dominance, 70% of the subjects demonstrated that their right eye being more myopic (dotted line in Fig. 3A) and 30% of the subjects showed their left eye being more myopic (dotted line in Fig. 3B) in all ranges of anisometropia. When the right eyes happened to be the sensory dominant eye as well, the effect of ocular dominance increased the percentage to 80%, but only in cases when the anisometropia was less than 3 D. When the left eye happened to be the dominant eye, the effect of ocular dominance increased the percentage to more than 40% albeit only when the anisometropia was less than 4 D. When the degree of anisometropia was very large, the eye with the smaller refractive error (less myopic) tended to be dominant. This brought down the percentage below 70% (from 80%) once anisometropia was beyond 3 D in subjects with a dominant right eye and brought the percentage down below 30% (from 40%) once anisometropia was beyond 4 D in the subjects with a dominant left eye (Fig. 3).

## Discussion

In this study, we showed that the effect of laterality shifted the baseline of which eye was more myopic from the level of 50–50 to the level of 70–30. The status of being the dominant eye further increased the chance of an eye being more myopic. The chance of being more myopic increased to 80% (from 70%) in the right-dominant eyes and to 40% (from 30%) in the left-dominant eyes. If we had not separated the right-dominant and left-dominant subjects, the existence of the dominant eye being more myopic would have been found in 60% of the subjects [(80 + 40)/2 = 60], which is statistically not far away from the 50–50 baseline.

### The right eye is more myopic

The finding of the right eye being more myopic is consistent with the results of several previous reports. Qiu *et al*. reported that in anisometropia less than 2 D, the right eye was more myopic in 70.1% of the subjects and the left eye was more myopic in 29.9% of the subjects^32^. In addition, Singh *et al*. reported that, in 31 anisometropic subjects, 24 (77%) showed greater myopia in the right eye^22^. Hong Kong children, aged between six and 8.5 years, and their parents were found to have a tendency toward more spherical myopia in the right eye (laterality) in myopic cases^4^. Chia *et al*.’s study also reported that there was a small but significantly longer axial length in the right eyes^23^. Regarding the reason as to why the right eye is more myopic in anisometropia, there has not been a clear explanation presented to date. Some studies reported that the right side of the face is significantly larger than the left side, and the accompanied asymmetry in eyeball difference may account for the finding that the right eye is more myopic^33^. Some researchers have speculated that the left hemisphere processes reading and comprehension, which are mostly done at near distance^32^. However, a large-scale study on more than 23,000 subjects suggested that near-distance work habits show no significant association with anisometropia^34^.

### After controlling for laterality, only sensory dominance is relevant

In anisometrope, the interocular difference in refractive errors causes one eye’s retinal image to defocus, either intermittently or constantly. Consequently, it results in reduced image clarity and contrast in that eye, and/or differences in retinal image size between the two eyes. In principle, the two eyes send different signals about a single object to the visual brain. It is the dissimilarity of the information from the eyes, rather than defocus, that causes damage to visual function^17^. Therefore, the relevant ocular dominance test should reflect the neural asymmetry. Among the different types of ocular dominance used, sighting dominance^23,25–28,30^ refers to the preferential use of one eye over the fellow eye when binocular fusion is impossible or binocular view is not practical. It is mainly related to the judgment of visual direction^35,36^, although such correlation may not be that strong^37^. In motor dominance, the nondominant eye is more likely to lose fixation at the near point of convergence. It probably reflects more about the state of the extraocular muscles with their innervational patterns than the preferential information process^38,39^. Sensory dominance occurs when the perception of a stimulus presented to one eye dominates the other in retinal rivalry conditions^40^. From this perspective, it is no wonder that only sensory dominance showed significant correlation after the effect of laterality has been controlled.

### Within certain range, the neural asymmetry compensates the optical asymmetry

It seems that maintaining relatively balanced signals sent by the two eyes is of immense importance to the development of the visual system. Therefore, during the development of the anisometropia, it is reasonable to believe that, as part of the visual pathway, the neural system may develop a certain asymmetry to counter or mitigate the interocular optical asymmetry and to bring the signals sent from the two eyes closer to balance. Indeed, some previous studies have indicated that ocular sensory dominance adapts to abnormal visual experience during the development^41,42^. In this present study, when the magnitude of anisometropia was less than moderate (4 D in the right and 4 D in the left), the neural asymmetry favoring the more myopic eye might mitigate the effect of the optical asymmetry and bring the signals sent from the two eyes close to balance. It is clear that ocular dominance is increasing with the magnitude of anisometropia (see Fig. 2B, bottom panel). However, when the magnitude of anisometropia goes beyond a certain threshold magnitude, the neural asymmetry may not be great enough to overcome the optical asymmetry and the probability of the myopic eye being dominant drops below the baseline level (70% in right and 30% in left).

This reversal of tend was not reported by previous studies. Relatively small sample sizes, in combination with a limited range of anisometropia tested, might explain this discrepancy. In Cheng *et al*.’s study, there were a total of 55 subjects. Thirty-three of them had anisometropia less than 1.75 D, 22 had it greater than 1.75 D, 18 had it greater than 2 D, and only one greater than 4.0 D^25^. In Vincent *et al*.’s study, there were a total of 34 subjects, with only five of them having anisometropia greater than 2.25 D^10^. In Qiu *et al*.’s study, there were 69 subjects with anisometropia less than 2.0 D and there were only 15 subjects with anisometropia greater than 2 D^32^. In our study, there were 49 subjects with anisometropia greater than 3.0 D, and among those, 14 of them were above 4 D.

### Better-than-expected visual functions in anisometropia

Recent studies reported that visual performance of anisometropes for acuity is better than what would be predicted strictly on the basis of retinal blur, and some of the anisometropes retained stereoacuity better than 40 arc sec.^17^. Those findings were considered as surprising based on the results from some previous studies. Here, we speculate on some potential explanations based on the findings from the present study. First, in normal subjects, stereopsis is degraded more by monocular blur than by both eyes being blurred^19,43^. However, given that the more myopic eye in an anisotrope tends to be the dominant one, the eventual signals sent from the two eyes to the visual cortex may not be that different. Second, the monocular acuity of an eye that is optically blurred is worse when the unblurred fellow eye is open than when it is occluded^44^. This suggests a suppressive effect from the clear eye. This study, again, was done with simulated anisometropia on normal subjects, whose neural asymmetry is small. In real anisometropes, the more myopic eye is often the dominant one and is less likely to be suppressed. Third, in anisometropic amblyopia, the amblyopic eye appears to be suppressed by the fellow eye^45^. This is not contradictory to our findings. As the magnitude of anisometropia increases, the less myopic eye becomes the dominant eye and the more myopic eye becomes the nondominant eye. Ocular dominance now amplifies the interocular optical difference, instead of mitigating it. Those speculations should be taken with caution and future studies are needed to test their validity. Alternatively, the residual stereopsis could be explained by either anisometropia showing up in a time that matters less in development, or by periods of clear vision, or by the availability of low spatial frequencies to both eyes^46^.

Although some anisometropes retained stereopsis, it would be interesting to test if they needed a much longer time to achieve optimal stereoacuity. Anisometropes tend to have large ODI and normal subjects who have large ODI need much longer time to see the fine stereopsis^47^.

### Limitation of the current study and future directions

Tong *et al*. found that the asymmetrically accelerated change in SE between the left and right eye is the cause of anisometropia. In the view that the more myopic eye is the dominant eye and is more sensitive to visual feedbacks^34^, we would expect that the more myopic eye should respond better to myopic control. A recent study confirmed this^34^. Chen *et al*. found that, in myopic anisometropic children who underwent orthokeratology lens treatment, the two eyes demonstrated different amounts of axial elongation and the more myopic eyes showed significantly less axial length elongation. The control groups, who were isomyopic children wearing matched orthokeratology lenses and anisometropic children wearing spectacles, had similar axial length elongation between the two eyes. Therefore, it was concluded that orthokeratology could reduce the amplitude of anisometropia in children primarily through stronger myopia control in the more myopic eye.

Because of the cross-sectional nature of this present study, we cannot be certain whether the greater neural asymmetry in anisometropia is an adaptation or if it is the cause of the increasing interocular optical asymmetry. Longitudinal studies that track the onset and progress of anisometropia and ocular dominance may shed more light on this question. Currently, a longitudinal study is under way in the authors’ lab to investigate if the establishment of ocular sensory dominance occurs prior to the emergence of anisometropia, and whether ocular dominance changes during the progress of anisometropia.

In conclusion, our study suggested that, in myopic anisometropes, which eye is more myopic is correlated with the interaction among laterality, ocular sensory dominance, and the magnitude of anisometropia.

## Methods and Materials

### Subjects

A total of 193 myopic anisometropic subjects, including 83 males and 110 females, were recruited from the clinic of the Department of Optometry of Wenzhou Medical University in Wenzhou, China. Participants’ age ranged from 10 years to 34 years with a mean age of 18.5 ± 6.2 years. Myopia was defined as a minus refractive error with the absolute value of spherical equivalent power greater than 0.50 D. Myopic anisometropia was defined as an interocular refractive error difference of greater than 1.00 D, with each eye being either emmetropic or myopic. The astigmatism in both eyes should be less than 1.00 D. Subjects were only included in the present study if they had a best corrected visual acuity of 20/20 or better at distance in each eye. Subjects were excluded from the study if they displayed any of the following conditions: latent hyperopia, strabismus or ptosis, prior ocular surgery, amblyopia, keratoconus, glaucoma, retinal diseases, optic disc abnormalities, optic neuropathy, or another disease that might affect best corrected visual acuity. The ethics board of the Wenzhou Medical University approved this study [approval number KYK[2015]15]. Prior to the start of the study, written informed consent was obtained from the subjects, and their parents or legal guardians if they were younger than 18 years of age, after all of the subjects’ questions and concerns were addressed. All procedures adhered to the tenets of the Declaration of Helsinki of the World Medical Association.

### Refraction

Cycloplegia was achieved with topical application of cyclopentolate hydrochloride eye drops (10 mg/ml, Alcon Inc., Fort Worth, USA), administrated as one drop every five minutes for a total of three times. Following a 20-minute break, each subject underwent objective refraction with a WAM 5500 autorefractor (Grand SEIKO Co. Ltd, Hiroshima, Japan). During the measurement, the tested eye was required to fixate on a bright spot (20 cd/m^2^) on a dark screen located six meters away, with the non-tested eye left open. Refractive errors [i.e., Spherical (S), Cylinder (C), axis (α)] were measured five times in each eye. SE and vector presentation of astigmatism J0 and J45 were calculated according to the following formulas: SE = S + C/2, J0 = (−C/2) × cos(2 × alpha), and J45 = (−C/2) × sin(2 × alpha), in which alpha represents the direction of the astigmatism. All 193 anisometropic subjects met this criterion.

### Sighting dominance

Sighting dominance was determined via the performance of the hole-in-the-card test. A subject held a card with both hands at arm’s length and viewed a target six meters away through a three-centimeter hole in the center of the card, with both eyes open. Each eye was then closed in turn to identify the dominant one. During this test, closing the dominant eye would lead to the disappearance of the target, while covering the nondominant eye would not. In each subject, the hole-in-the-card test was performed three times. The eye demonstrated as dominant for two or more times was considered as the dominant eye.

### Motor dominance

The convergence near-point test was used to determine the motor dominance. For the test, the subject was asked to fixate on the tip of a small stick moving toward the nasal bridge with both eyes open. During this process, the examiner watched if divergence occurred in the eyes. In cases where a deviation could be clearly identified, the eye that deviated first was designated as the nondominant eye and the eye that remained fixated was designated as the dominant one. Cases were classified as “undetermined” when it was difficult to decide which eye deviated first.

### Sensory eye dominance measurement

**Figure 4:**
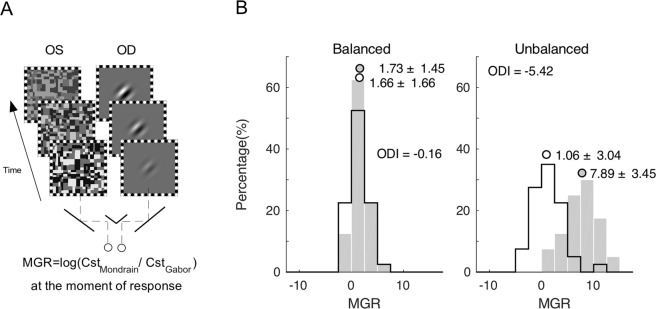
The method to measure ocular dominance. (**A**) The Mondrian/Gabor contrast ratio (MGR = Log (Cst_Mondrian_/Cst_Gabor_) was calculated at the time of response in each trial and each eye was tested 50 times. (**B**) Examples of subjects with balanced eyes (left panel) and unbalanced eyes (right panel) are shown here. The open and filled bars represent the right and left eyes’ MGR values, respectively. The open and filled circles represent the right and left eyes’ median values of MGR, respectively.

Ocular sensory dominance was measured with the continuous flashing technique^48^. Stimuli were presented in the center of a CRT monitor (1024 × 768 resolution, 100 Hz; Richardson Electronics, LaFox, IL, USA) against a uniform background (mean luminance 50 cd/m^2^) and viewed at a distance of 60 cm with a chin rest. The dynamic Mondrian patterns subtended 4.3° × 4.3°, with individual elements extended by 0.154°. The target stimulus was a Gabor patch tilted 45° toward either the right or the left (spatial frequency = 1 cycle/degree, spatial extension 1.9°). The black and white strokes that framed the Mondrian and Gabor patches were 0.33° in width and were used to help achieve binocular fusion. Mirrors were used to present the Mondrian and target stimuli dichoptically. Each eye exclusively viewed one of the two stimuli during a given trial. The eyes viewing the dynamic Mondrian and target stimuli were counterbalanced and randomized across trials (Fig. 4A). The experiment was programmed in MATLAB (version 2012Rb from The MathWorks, Inc., Natick, MA) and the Psychophysics Toolbox, (version 3)^49,50^.

At the beginning of a trial, the tested eye viewed the target Gabor patch at 0% contrast and the fellow eye viewed a full contrast Mondrian pattern. During a trial, the contrast of the Gabor patch linearly increased at a rate of 1% every 100 ms, while the contrast of Mondrian patterns linearly decreased at the same rate. The subjects were tasked with reporting, by pressing one of two keys, that time at which the obliquely oriented Gabor patches were detected. A trial terminated once this action was made. For each trial, the log ratio of Mondrian to Gabor’s contrasts (MGR) at the moment of response was computed. A higher ratio signified a greater quantitative measure of the ocular sensory dominance of that eye.

Subjects performed 10 practice trials prior to starting 50 experimental trials. A rank-sum test was used to compare the 50 MGRs collected for each eye. The Z-value, which was the interocular difference in mean values normalized by the standard deviations of values from both eyes, was used as the ODI to quantify a subject’s overall degree of ocular dominance. A positive value of ODI indicated that the right eye was stronger, while a negative value of ODI indicated that the left eye was stronger. An ODI with an absolute value of 2, which corresponded to a p value of 0.05 for the sample size of 50, was selected as the significance level. A subject with an absolute value of ODI larger than 2 was regarded as having clear ocular dominance (Fig. 4B, right panel). On the other hand, a subject with an absolute ODI value of less than 2 was regarded as having balanced eyes (i.e., Fig. 4B, left panel).

All ocular dominance tests were conducted by the first author, an ophthalmologist, who was masked to the refractive status of the subjects.

### Data analyses and statistics

Statistical analyses were performed with the R programming package (version 3.3.1; The R Foundation, Vienna, Austria). Mean, median, and standard deviation were used for descriptive purposes. A Wilcoxon rank-sum test was performed to test the significant differences in refractive error between the left and right eyes. Pearson’s r was used to calculate the correlation between the amplitude of anisometropia and ODI. Multiple logistic regression was used to test if the probability of an eye being the more myopic one, between the two eyes, was significantly associated with laterality, being the dominant eye, and the amount of anisometropia. A p-value of less than 0.05 was considered to be statistically significant.

## References

[1] Weale RA (2002). On the age-related prevalence of anisometropia. Ophthalmic Res.

[2] Liang YB (2009). Refractive errors in a rural Chinese adult population the Handan eye study. Ophthalmology.

[3] Tanlamai T, Goss DA (1979). Prevalence of monocular amblyopia among anisometropes. Am J Optom Physiol Opt.

[4] Goldschmidt E, Lyhne N, Lam CS (2004). Ocular anisometropia and laterality. Acta Ophthalmol Scand.

[5] Tong L, Saw SM, Chia KS, Tan D (2004). Anisometropia in Singapore school children. Am J Ophthalmol.

[6] Borchert M (2010). Anisometropia in Hispanic and african american infants and young children the multi-ethnic pediatric eye disease study. Ophthalmology.

[7] Deng L, Gwiazda JE (2012). Anisometropia in children from infancy to 15 years. Invest Ophthalmol Vis Sci.

[8] Tong L, Chan YH, Gazzard G, Tan D, Saw SM (2006). Longitudinal study of anisometropia in Singaporean school children. Invest Ophthalmol Vis Sci.

[9] Kwan WC, Yip SP, Yap MK (2009). Monochromatic aberrations of the human eye and myopia. Clin Exp Optom.

[10] Vincent SJ, Collins MJ, Read SA, Carney LG, Yap MK (2011). Interocular symmetry in myopic anisometropia. Optom Vis Sci.

[11] Lee SM, Edwards MH (2000). Intraocular pressure in anisometropic children. Optom Vis Sci.

[12] Lombardo M, Lombardo G, Serrao S (2006). Interocular high-order corneal wavefront aberration symmetry. J Opt Soc Am A Opt Image Sci Vis.

[13] Yibin T., Janice T. and Wildsoet, C F. Optical and biometric bases of anisometropia. *Invest Ophthalmol Vis Sci* **47**, E-abstract 3677 (2006).

[14] Vincent SJ, Collins MJ, Read SA, Carney LG (2014). Myopic anisometropia: ocular characteristics and aetiological considerations. Clin Exp Optom.

[15] Huynh SC (2006). Prevalence and associations of anisometropia and aniso-astigmatism in a population based sample of 6 year old children. Br J Ophthalmol.

[16] O’Donoghue L (2013). Profile of anisometropia and aniso-astigmatism in children: prevalence and association with age, ocular biometric measures, and refractive status. Invest Ophthalmol Vis Sci.

[17] Levi DM, McKee SP, Movshon JA (2011). Visual deficits in anisometropia. Vision Res.

[18] Ong J, Burley WS (1972). Effect of induced anisometropia on depth perception. Am J Optom Arch Am Acad Optom.

[19] Westheimer G, McKee SP (1980). Stereogram design for testing local stereopsis. Invest Ophthalmol Vis Sci.

[20] Mapp AP, Ono H, Barbeito R (2003). What does the dominant eye dominate? A brief and somewhat contentious review. Percept Psychophys.

[21] Porac C, Coren S (1976). The dominant eye. Psychol Bull.

[22] Singh N, Rohatgi J, Kumar V (2017). A Prospective Study of Anterior Segment Ocular Parameters in Anisometropia. Korean J Ophthalmol.

[23] Chia A (2007). Ocular dominance, laterality, and refraction in Singaporean children. Invest Ophthalmol Vis Sci.

[24] Tekin K, Cankurtaran V, Inanc M, Sekeroglu MA, Yilmazbas P (2017). Effect of myopic anisometropia on anterior and posterior ocular segment parameters. Int Ophthalmol.

[25] Cheng CY, Yen MY, Lin HY, Hsia WW, Hsu WM (2004). Association of ocular dominance and anisometropic myopia. Invest Ophthalmol Vis Sci.

[26] Ito M (2013). Association between ocular dominance and refractive asymmetry. J Refract Surg.

[27] Linke SJ (2011). Association between ocular dominance and spherical/astigmatic anisometropia, age, and sex: analysis of 10,264 myopic individuals. Invest Ophthalmol Vis Sci.

[28] Linke SJ, Baviera J, Richard G, Katz T (2012). Association between ocular dominance and spherical/astigmatic anisometropia, age, and sex: analysis of 1274 hyperopic individuals. Invest Ophthalmol Vis Sci.

[29] Eser I, Durrie DS, Schwendeman F, Stahl JE (2008). Association between ocular dominance and refraction. J Refract Surg.

[30] Yang Z (2008). Association of ocular dominance and myopia development: a 2-year longitudinal study. Invest Ophthalmol Vis Sci.

[31] Jiang F (2015). Association between Ocular Sensory Dominance and Refractive Error Asymmetry. PLoS One.

[32] Qiu KL (2004). F. The dominant eye in myopes. Chinese Journal of Optometry and Ophthalmology.

[33] Ferrario VF, Sforza C, Miani A, Serrao G (1995). A three-dimensional evaluation of human facial asymmetry. J Anat.

[34] Lee CW (2017). Prevalence and association of refractive anisometropia with near work habits among young schoolchildren: The evidence from a population-based study. PLoS One.

[35] Money J (1972). Studies on the function of sighting dominance. Q J Exp Psychol.

[36] Reiss MR (1997). Ocular dominance: some family data. Laterality.

[37] Sridhar D, Bedell HE (2011). Relative contributions of the two eyes to perceived egocentric visual direction in normal binocular vision. Vision Res.

[38] Horng JL, Semmlow JL, Hung GK, Ciuffreda KJ (1998). Dynamic asymmetries in disparity convergence eye movements. Vision Res.

[39] Kawata H, Ohtsuka K (2001). Dynamic asymmetries in convergence eye movements under natural viewing conditions. Jpn J Ophthalmol.

[40] Evans BJ (2007). Monovision: a review. Ophthalmic Physiol Opt.

[41] Porac C, Coren S (1975). Suppressive processes in binocular vision: ocular dominance and amblyopia. Am J Optom Physiol Opt.

[42] Toch H (1960). Can eye dominance be trained?. Perceptual & Motor Skills.

[43] Legge GE, Gu YC (1989). Stereopsis and contrast. Vision Res.

[44] Simpson T (1991). The suppression effect of simulated anisometropia. Ophthalmic Physiol Opt.

[45] Hess RF, Pointer JS (1985). Differences in the neural basis of human amblyopia: the distribution of the anomaly across the visual field. Vision Res.

[46] Holopigian K, Blake R, Greenwald MJ (1986). Selective losses in binocular vision in anisometropic amblyopes. Vision Res.

[47] Wu H (2018). Balanced Eyes See Stereopsis More Quickly, but Not More Finely. Invest Ophthalmol Vis Sci.

[48] Yang E, Blake R, McDonald JE (2010). A new interocular suppression technique for measuring sensory eye dominance. Invest Ophthalmol Vis Sci.

[49] Brainard DH (1997). The Psychophysics Toolbox. Spat Vis.

[50] Pelli DG (1997). The VideoToolbox software for visual psychophysics: transforming numbers into movies. Spat Vis.

